# The Brief Experiential Avoidance Questionnaire: Validation of the French Version in Non-clinical Adults

**DOI:** 10.5334/pb.1256

**Published:** 2024-10-04

**Authors:** Esin Er, Aurélie Wagener, Anne-Marie Étienne, Marie Vander Haegen

**Affiliations:** 1 Research Unit for a life-Course perspective on Health & Education –RUCHE, Faculty of Psychology, Speech and Language Therapy and Educational Sciences, University of Liège, Belgium; 2 Laboratoire de Psychopathologie et Processus de la santé, Universitéde Paris, France

**Keywords:** experiential avoidance, BEAQ, French, validation

## Abstract

**Introduction:**

Various studies indicate the role of experiential avoidance (EA) in the onset and maintenance of mental health disorders such as depression or anxiety disorders. The Brief Experiential Avoidance Questionnaire (BEAQ) is a quick tool to assess EA.

**Objective:**

This study aimed to translate the BEAQ into French and to validate it in a sample of non-clinical adults.

**Method:**

The BEAQ was translated from English into French using the translation and back-translation technique. The translated questionnaire was submitted to 93 psychology students to identify unclear/ambiguous items. Then, the final French and the original versions of the BEAQ were administered to 64 bilingual individuals two weeks apart to assess the scale’s content validity and test-retest reliability. Finally, the BEAQ-French and other scales were administered to 580 non-clinical adults to assess its factor structure and its convergent and discriminant validity.

**Results:**

Results showed no significant difference between the English and French versions of the BEAQ. The BEAQ demonstrated high internal consistency and good test-retest reliability. Confirmatory factor analyses did not support the one-factor structure of the BEAQ. Exploratory factor analyses revealed a two-factor structure. The BEAQ presented satisfactory convergent and discriminant validity with related measures and measures of neuroticism, negative affect and mental health problems.

**Conclusion:**

The results suggest that the BEAQ-French is a reliable tool for assessing EA. Unlike the original version, the BEAQ-French exhibits a two-factor rather than a one-factor structure. Further research is needed to confirm this two-factor structure and to assess more precisely the convergent validity of the scale.

Experiential avoidance (EA) is defined as *“the phenomenon that occurs when a person is unwilling to remain in contact with particular private experiences (e.g., bodily sensations, emotions, thoughts, memories, behavioral predispositions) and takes steps to alter the form or frequency of these events and the contexts that occasion them”* (Hayes et al., 1996, p. 1154). The actions taken to modify these experiences (or the events, the situations that trigger them) include avoidance and escape in all their forms (Chawla & Ostafin, 2007; Davis et al., 2022; Hayes, 2004) as long as they are initiated to modify internal experiences and the contexts that trigger them (Hayes, 2004; Hayes et al., 1996). When it is rigid and chronic, EA has a deleterious effect: it enables people to suppress or reduce the intensity of unpleasant experiences in the short term, but it is associated with a recurrence of unpleasant emotions, thoughts, memories and reinforces their frequency and intensity in the long term (Bardeen, 2015; Hayes, 2004).

EA is considered by some authors as a trait-like characteristic and is generally measured in this way (Akbari et al., 2022; Gámez et al., 2011; Kirk et al., 2021; Lewis et al., 2023; Spinhoven et al., 2014). Many studies, including longitudinal research studies, indicate that it promotes the use of maladaptive avoidance strategies (e.g., problematic alcohol use, substance abuse, self-harm) and emphasize its role in the onset and maintenance of mental health disorders (e.g., depression, anxiety, compulsive behaviors, eating disorders, alcohol use disorder) (Chawla & Ostafin, 2007; Den Ouden et al., 2020; Haywood et al., 2023; Luoma et al., 2020; Spinhoven et al., 2014). Moreover, high levels of EA are associated with poorer quality of life and lower satisfaction with life (Gámez et al., 2011; Schaeuffele et al., 2022). Some recent research also points to a relation between EA and worse physical health. One explanation is that EA is associated with the use of maladaptive strategies to avoid internal experiences (e.g., overeating, overdrinking) and these strategies have negative consequences for physical health. Furthermore, chronically avoiding thoughts and emotions increases distress and physiological dysregulation, which is a predictor of physical health problems (Berghoff et al., 2017; Blakey et al., 2021).

Given its involvement in a wide variety of disorders, EA is considered a *“transdiagnostic risk factor”* (Spinhoven et al., 2014, p. 841) and is an explicit therapeutic target and/or a mechanism of change (i.e., a mediator of the effect) in third-wave therapies such as acceptance and commitment therapy, dialectical behavior therapy and mindfulness-based interventions (Cavicchioli et al., 2020; Gámez et al., 2014; Hayes & Wilson, 1994; He et al., 2023; McCluskey et al., 2022; Neacsiu et al., 2014; Yela et al., 2022; Yela et al., 2020). Several studies indicate that a reduction in EA levels is associated with a decrease in the severity of certain mental health problems (e.g., Ellis & Rufino, 2016; Eustis et al., 2016). For instance, Yela et al. (2022) showed that reduced EA scores after a mindfulness and self-compassion training explained changes in anxiety, depression and well-being scores. Another study showed that, in a sample of hospitalized patients, a reduction in EA during treatment was associated with a decrease in suicidal ideation. Importantly, the relation between the reductions in EA and in suicidal ideation was independent of changes in hopelessness and depression scores (Ellis & Rufino, 2016).

The Acceptance and Action Questionnaire (AAQ) and its revised version, the AAQ-II (Bond et al., 2011; Hayes et al., 2004) are the most widely used tools to measure EA. However, they have some limitations. First, the AAQ-II has been reconceptualized as a measure of psychological flexibility, which is a much broader concept than EA (Bond et al., 2011). It is defined as *“the ability to persist or change the course of an action, even in the presence of unpleasant thoughts, sensations and emotions, in order to move in the direction of what is important to the person, that is to say, his personal values”* (Dionne et al., 2014, p. 115). According to the ACT model of psychological flexibility (i.e., Hexaflex), six processes contribute to the loss of psychological flexibility (i.e., cognitive fusion, experiential avoidance, thoughts focused on the future and the past, the conceptualized self, lack of clarity on values, and inaction, impulsivity, and avoidance of situations) (see Hayes et al., 2011 for a detailed description of the model). Since EA is just one of these processes, it does not consist in an interchangeable concept with psychological flexibility (Hayes et al., 2011). Second, several studies indicate that the AAQ-II has insufficient discriminant validity since its scores are more strongly correlated with measures of negative affect and neuroticism than with other measures of avoidance or acceptance (Gámez et al., 2014; Gámez et al., 2011; Rochefort et al., 2018). This made some researchers suggest that the AAQ-II is more a measure of distress, neuroticism or negative affect, than of EA or psychological flexibility (Rochefort et al., 2018; Tyndall et al., 2019; Wolgast, 2014).

In response to the limitations of the AAQ-II, Gámez et al. (2011) developed the Multidimensional Experiential Avoidance Questionnaire (MEAQ) as a specific measure of EA. Unlike the AAQ-II, which has a one-factor structure, the MEAQ is a multidimensional measure of EA. It includes six subscales and 62 items: (1) Behavioral Avoidance (*“overt and situational avoidance of physical discomfort and distress”*); (2) Distress Aversion (*“negative evaluations or attitudes toward distress, non-acceptance of distress”*); (3) Procrastination (*“delaying anticipated distress”*); (4) Distraction/Suppression (*“attempts to ignore or suppress distress”*); (5) Repression/Denial (*“distancing and dissociating from distress, lack of distress awareness”*); and (6) Distress Endurance (*“willingness to behave effectively in the face of distress”*) (Gámez et al., 2011, p. 700). The multidimensionality of the MEAQ reflects the complexity of the EA construct and highlights that it may operate differently in different individuals and that individuals presenting high levels of EA may exhibit distinct facets of EA (Gámez et al., 2011; Kirk et al., 2021). For example, an individual may primarily manifest the behavioral avoidance dimension of EA, showing a tendency to leave or avoid situations resulting in unpleasant internal experiences, while in another individual, the cognitive avoidance dimension may be dominant, taking the form of attempts to suppress unpleasant thoughts or distract oneself from difficult internal experiences.

Unlike the AAQ-II, the MEAQ demonstrated satisfactory discriminant validity with respect to measures of negative affect and neuroticism in student and patient populations (Gámez et al., 2011). However, the MEAQ’s length may be a barrier to its use in clinical practice and research. Therefore, briefer versions of the MEAQ have been developed, one with 30 items (Sahdra et al., 2016) and another with 15 items (Gámez et al., 2014). For pragmatic reasons, this article is focused on the briefest version of the original MEAQ: the Brief Experiential Avoidance Questionnaire (BEAQ). To create this scale, Gámez et al., (2014) selected among the MEAQ items, those presenting high loadings on a single common factor and they ensured that each MEAQ subscale was represented by at least one item in the final shorter scale. The BEAQ comprises 15 items, four of which belong to the “Behavioral Avoidance” subscale of the MEAQ, four to the “Distress Aversion” subscale, two to the “Suppression/Distraction” subscale, two to the “Repression/Denial” subscale, two to the “Procrastination” subscale and one to the “Distress Endurance” subscale. It presents satisfactory discriminant and convergent validity in student, patient and community populations, similar to the MEAQ (Gámez et al., 2014). The BEAQ is claimed to have a one-factor structure. It has already been validated in Spanish, German, Chinese and Polish (Cao et al., 2021; Schaeuffele et al., 2022; Vázquez-Morejón et al., 2019; Wardęszkiewicz & Holas, 2024). However, results from several of these validation studies did not support a one-factor structure and showed that a two-factor structure or even a five-factor structure in the German version (with the original MEAQ subscales, except distress endurance) fit the data better (Cao et al., 2021; Schaeuffele et al., 2022; Wardęszkiewicz & Holas, 2024).

Given the limitations of the AAQ-II and the interest of a brief measure of EA for clinical practice and research, the aim of the current study was to validate the French version of the BEAQ. This is a first study of the psychometric properties and the factor structure of the French version of this scale.

## Methods

The BEAQ was translated and validated in French in several stages. First, the scale was translated and submitted to a sample of psychology students to identify unclear or ambiguous items. Then the internal consistency, content validity and test-retest reliability of the translated version were examined in a sample of bilingual participants. Finally, the factor structure and the relationship between the BEAQ and other scales, including mental health and affect measures, were studied.

### French translation of the BEAQ and preliminary assessment

As presented above, the first step involved translating the scale and identifying unclear or ambiguous items. Permission was obtained from the authors of the BEAQ to conduct the French translation and the validation study of the scale. The BEAQ was translated using the translation and back-translation technique (Vallerand, 1989). First, the items of the scale were translated from English into French by one of the authors (MVH); then they were back-translated from French to English by a bilingual researcher who holds a doctorate in psychology. The two versions were then compared using a committee approach (Vallerand, 1989). Discrepancies between the English and French versions were discussed, and corrections made.

The pre-final version of the scale was then submitted to a sample of 93 psychology students, who assessed all the items of the translated version on a “clear-unclear” binary scale (Krings et al., 2021; Vallerand, 1989). Each time they considered an item “unclear”, participants were asked to explain why and suggest changes. It had been decided beforehand that the items evaluated as “unclear” by at least 20% of the participants would be re-analyzed and reformulated (Krings et al., 2021; Vallerand, 1989). None of the items reached this percentage (see Supplementary Material Section A for the French version of the Brief Experiential Avoidance Questionnaire).

The second validation step aimed to examine the internal consistency, content validity and test-retest reliability of the translated version. Following an approach proposed by Haccoun (1987) and applied in several studies (e.g. Krings et al., 2021; Rault & Décamps, 2022; Wagener & Blairy, 2015), the English and French versions of the BEAQ were administered to 64 English-French bilingual adults (44 women and 20 men), at 15-day intervals to test the scale’s internal consistency and temporal stability. Before answering the questionnaires, participants first had to assess on a scale of 1 (very little) to 4 (perfectly) their ability to read, write, understand a conversation and speak in English and French. Participants who scored less than 12/16 for English or French were excluded from the sample. All bilingual participants were from Belgium. Among the 64 adults included in the sample, 36 completed the French questionnaire first and 28 completed the English questionnaire first. After assumption checks were conducted, a Student’s *t* test was performed to compare the total scores on the two versions of the BEAQ and showed no significant difference between the total scores on the English and French versions of the BEAQ (*t* (63) = 0.08, *p* = .94). Moreover, all items in English and French were positively and significantly correlated with each other (all *r* > .39, *p* < .01). Both *t*-test’s and correlations’ results led to reject the hypothesis of non-equivalence between the English and the French versions of the BEAQ. Thus, we can conclude that there is no significant difference between these two versions of the BEAQ in our sample. The intraclass correlation coefficient between the total scores on the two versions of the BEAQ administered two weeks apart was .88 (*p* < .001), which underscores the tool’s good test-retest reliability. Cronbach’s alpha for the BEAQ-French was .87.

The resulting French version of the scale was then administered to a new sample to study its factor structure and construct validity. This constituted the third and final validation stage. The information and results presented in the following sections relate to this third validation stage. All of these procedures were approved by the Ethics Committee of the Faculty of Psychology of the University of Liège (no. 2223-018).

### Participants and procedure

Participants were recruited through links posted on social media and the university’s messaging system. An information and consent form preceded the questionnaires. Responses were anonymous and were collected through the online survey system of the University of Liège (secure software specially designed for online data collection). There was no reward for participation.

In order to maximize the sample size, the only inclusion criterion for this third phase of the study was that participants had to be 18 years or older. Overall, 622 individuals completed the online questionnaires, 42 of whom were excluded due to incomplete responses. The final sample included 580 participants (455 women and 125 men) whose responses were complete for all questionnaires. Only the responses of these 580 participants were used in the analyses. Participants’ sociodemographic characteristics are reported in Table 1.

**Table 1:** Sociodemographic characteristics of the participants (n = 580).


CHARACTERISTIC	M (SD) OR F (%)

Age	40.06 (13.85)

Gender	

Female	455 (78.4%)

Male	125 (21.6%)

Education level	

Less than high school	26 (4.5%)

High school	83 (14.3%)

Short-course higher education	167 (28.8%)

Long-course higher education	173 (29.8%)

Postgraduate degree	131 (22.6%)

Professional status	

Student	70 (12.1%)

Worker	389 (67.1%)

Independent	29 (5%)

On sick leave	13 (2.2%)

Incapacity for employment	12 (2.1%)

Unemployed	13 (2.2%)

Stay-at-home parent	9 (1.6%)

Retired	34 (5.9%)

Other	11 (1.9%)


### Measures

Participants completed different questionnaires to study the BEAQ’s convergent and discriminant validity. As mentioned earlier, to be considered a valid measure of EA, the BEAQ should correlate more strongly with related constructs such as psychological flexibility than with measures of neuroticism, negative affect and psychopathology (Boateng et al., 2018; Krabbe, 2017). Therefore, the questionnaires included the BEAQ, a measure of psychological flexibility and measures of negative affect, neuroticism and mental health problems. The choice of these specific scales was based on previous BEAQ validation studies (Cao et al., 2021; Gámez et al., 2014; Schaeuffele et al., 2022; Vázquez-Morejón et al., 2019; Wardęszkiewicz & Holas, 2024) and practical considerations (e.g., the length of the scales).

#### The sociodemographic questionnaire

A sociodemographic questionnaire included questions on participants’ gender, age, level of education and professional status.

#### The Brief Experiential Avoidance Questionnaire (BEAQ; Gámez et al., 2014)

The BEAQ is a measure of EA that comprises 15 items, which participants respond to on a Likert scale ranging from 1 (“strongly disagree”) to 6 (“strongly agree”). The instructions were as follows: “Please indicate to what extent you agree or disagree with each of the following statements.” An example item is: “I quickly cut short any situation that makes me uncomfortable.” The score for Item 6 must be reversed. Scores range from 15 to 90, with higher scores indicating higher levels of EA. The original version of the BEAQ demonstrated good internal consistency (mean Cronbach’s alpha of .84).

#### The Acceptance and Action Questionnaire-II (AAQ-II; Bond et al., 2011; French: Monestès et al., 2009)

The AAQ-II is a one-dimensional measure of psychological flexibility, commonly used to measure EA. The French version of the AAQ-II includes 10 items rated on a Likert scale from 1 (“never true”) to 7 (“always true”). The instructions were as follows: “Here is a list of affirmations. Please rate how true each statement is for you by checking the number that corresponds to your answer.” It includes statements such as “I worry about not being able to control my worries and feelings”; “My painful memories prevent me from having a fulfilling life”. The scores for items 2, 3, 4, 5, 7, 8 and 9 are reversed. Scores range between 10 and 70, with higher scores indicating higher levels of psychological flexibility and lower levels of EA. The mean Cronbach’s alpha of the French version of the AAQ-II across samples was .82 (Monestès et al., 2009). Cronbach’s alpha for the present study was .86.

#### The Positive and Negative Affect Schedule (PANAS; Watson et al., 1988; French: Caci & Baylé, 2007)

This scale assesses positive and negative affectivity using 20 items divided into two 10-item subscales. These evaluate the frequency of different positive (e.g., interested, excited, etc.) and negative (e.g., scared, upset, etc.) affects during the past week. Items are rated on a Likert scale ranging from 1 (“very little or not at all”) to 6 (“very often”). Instructions were as follows: “Indicate the extent you have felt this way over the past week.” Examples of investigated affective states are “interested”, “strong”, “enthusiastic” for the positive ones and “distressed”, “guilty”, “scared” for the negative ones. To our knowledge, there is no information on the internal consistency of the French version of the PANAS from Caci and Baylé (2007). In the present study, Cronbach’s alpha was .86 for the Positive Affect subscale and .90 for the Negative Affect subscale.

#### The Satisfaction With Life Scale (SWLS; Diener et al., 1985; French: Blais et al., 1989)

The SWLS is a one-dimensional measure of satisfaction with life. It consists of five items rated on a Likert scale ranging from 1 (“strongly disagree”) to 7 (“strongly agree”). Instructions were as follows: “Below are five statements with which you may agree or disagree. Using the 1–7 scale below, indicate your agreement with each item. Please be open and honest in your responding.” An example item is: “In most ways my life is close to my ideal.” The French version of the SWLS demonstrates good internal consistency (mean Cronbach’s alpha of .81). Cronbach’s alpha for the present study was .86.

#### The Short Form of the Big Five Inventory (BFI-10; Rammstedt, 2007; French: Courtois et al., 2020)

The BFI-10 assesses the personality domains highlighted in the Big Five Factors personality model: extraversion, neuroticism, conscientiousness, agreeableness, and openness. It is a short version of the longer 44-item Big Five Inventory (John et al., 1991). It comprises two items for each personality dimension, with one of the two items being reversed. Items are rated on a 5-point Likert scale ranging from 1 (“disagree strongly”) to 5 (“agree strongly”). Instructions were as follows: “How well do the following statements describe your personality?” It includes statements such as “I see myself as someone who is relaxed, handles stress well” (from the Neuroticism subscale). Most BFI-10 subscales in the French validation study had low Cronbach’s alpha coefficients (below .70 for all subscales except Neuroticism). Nevertheless, we used this scale since: (1) the low alpha coefficients might be explained by the small number of items per subscale (Courtois et al., 2020); (2) the Neuroticism subscale was the most important for the study of the construct validity of the BEAQ and this specific subscale presented satisfactory internal consistency; and (3) because of its very short administration time. Cronbach’s Alpha for the present research was .71 for the Extraversion subscale, .10 for the Agreeableness subscale, .49 for the Conscientiousness subscale, .81 for the Neuroticism subscale and .28 for the Openness subscale.

#### The Hospital Anxiety and Depression Scale (HADS; Zigmond & Snaith, 1983; French: Bocéréan & Dupret, 2014)

The HADS is a scale for detecting anxiety and depressive disorders. It includes 14 items, 7 measuring anxiety symptoms (HADS-A) and 7 measuring depressive symptoms (HADS-D). The items are rated on a Likert scale from 0 to 3, with the total score ranging between 0 and 42. Instructions were as follows: “Please read each item below and check the answer that comes closest to how you have been feeling this past week. Give an immediate response and be dissuaded from thinking too long about the answers.” It includes items such as “I still enjoy the things I used to enjoy”; “Worrying thoughts go through my mind”. The French version of this tool presents good internal consistency, with a Cronbach’s alpha coefficient of .78 for the Depression subscale and .81 for the Anxiety subscale. Cronbach’s alpha for the present study was .76 for the Depression and .81 for the Anxiety subscale. The different questionnaires used were presented to the participants in the following order: (1) sociodemographic questionnaire, (2) BEAQ, (3) SWLS, (4) AAQ-II, (5) HADS, (6) BFI-10, and (7) PANAS.

### Statistical analysis

Exploratory and confirmatory factor analyses were performed to examine the factor structure of the BEAQ-French. First, a confirmatory factor analysis was performed to test the one-factor structure of the BEAQ. The model fit was determined based on four indices (Schweizer, 2010): (1) a model χ^2^ less than 3, (2) a root mean square error of the approximation (RMSEA) less than or equal to .08, (3) a Bentler Comparative Fit Index (CFI) greater than or equal to .90, and (4) a standardized root mean square residual (SRMR) less than or equal to .08.

Before beginning the research, it was planned that, if the confirmatory factor analysis did not support the one-factor structure of the BEAQ, an exploratory factor analysis would be carried out. Since the one-factor structure demonstrated an insufficient level of fit to the data, exploratory factor analyses were performed. A principal component analysis with oblimin rotation was carried out. The Bartlett sphericity test and Kaiser-Meyer-Olkin (KMO) test were used to determine if the data were adequate for factor analysis (Fabrigar et al., 1999; Shrestha, 2021). Data were considered adequate if the sphericity test was statistically significant with a *p*-value < .01 and the KMO statistic was greater than or equal to .80 (a KMO less than .50 being unacceptable) (Field, 2009; Shrestha, 2021). As in several other validation studies, the number of factors retained was determined based on the scree plot (Cao et al., 2021; Cattell & Vogelmann, 1977; Vander Haegen et al., 2022). Only items with a minimum load value of .30 were retained (Field, 2009).

Cronbach’s alpha was used to assess the scale’s internal consistency. Floor and ceiling effects were evaluated, and construct validity was assessed based on Pearson correlations between BEAQ scores and AAQ-II, PANAS, SWLS, BFI-10 and HADS scores. The significance level was set to *p* < .05. All the statistical analyses were conducted using IBM SPSS Statistics 28.0, except for the factor analyses which were conducted with Jamovi 2.3.21 software.

## Results

### Analysis of the BEAQ-French’s factor structure

The results of the confirmatory factor analysis did not support a one-factor structure for the BEAQ, since fit indices indicated that a one-factor solution did not fit adequately (the normalized χ² = 6.05, RMSEA of .09, 90% CI [.09; .10], CFI = .80, and SRMR = .06). In response to the poor fit of the one-factor model, an exploratory factor analysis was conducted on the data using principal component analysis with an oblimin rotation (n = 580). The KMO statistic was .87, which showed sampling adequacy for factor analysis (Kaiser & Rice, 1974). None of the items presented a KMO index below .60. All were above .82 except for items 4, 6 and 9 (KMO indices of .70, .67 and .77, respectively). The Bartlett sphericity test was statistically significant (χ² = 2300; df = 105; *p* < .001), indicating that the inter-item correlations were not all equal to zero and therefore a principal component analysis could be performed.

The results of the principal component analysis indicated that four factors had an eigenvalue greater than 1. Together, these four factors explained 56.09% of the variance. A scree plot was produced in order to determine the appropriate number of factors to be retained; it indicated that only two factors should be retained (see Supplementary Material Section B for the scree plot). These two factors explained 40.63% of the variance.

Table 2 presents the factor loadings of the 15 items of the BEAQ-French on the two factors retained. The component matrix indicated that 10 items loaded more strongly on the first factor and five items loaded more strongly on the second factor. The first factor was labeled “Avoidance Tendency” (items 1, 2, 3, 7, 8, 11, 12, 13, 14 and 15); a higher score on this subscale indicates higher levels of avoidance. The second factor was labeled “Emotional Unawareness and Inaction” (items 4, 5, 6, 9 and 10); higher scores on this subscale indicate higher levels of emotional unawareness and inaction. The loadings obtained for the 10 items on the first factor varied between .40 and .81. Loadings ranged between .37 and .73 for the second factor. The first subscale included all the items from the original “Distress Aversion”, “Behavioral Avoidance” and “Distraction/Suppression” subscales of the MEAQ while the second subscale included the items associated with the “Repression/Denial”, “Procrastination”, and “Distress Endurance” subscales (Gámez et al., 2011).

**Table 2:** Factor loadings of the BEAQ items.


BEAQ ITEMS	M	SD	FACTOR		UNIQUENESS

			1	2	

1. The key to a good life is never feeling pain.	3.04	1.55	**.66**	–.20	.63

2. I’m quick to leave any situation that makes me feel uneasy.	3.83	1.42	**.49**	.04	.75

3. When unpleasant memories come to me, I try to put them out of my mind.	4.26	1.53	**.50**	–.08	.78

4. I feel disconnected from my emotions.	2.27	1.5	–.02	**.65**	.59

5. I won’t do something until I absolutely have to.	2.32	1.43	.11	**.63**	.54

6. Fear or anxiety won’t stop me from doing something important.	2.75	1.63	–.17	**.50**	.79

7. I would give up a lot not to feel bad.	3.22	1.44	**.51**	.26	.58

8. I rarely do something if there is a chance that it will upset me.	3.08	1.4	**.56**	.19	.58

9. It’s hard for me to know what I’m feeling.	2.63	1.59	.00	**.73**	.47

10. I try to put off unpleasant tasks for as long as possible.	3.69	1.56	.26	**.37**	.72

11. I go out of my way to avoid uncomfortable situations.	4.02	1.38	**.70**	.05	.48

12. One of my big goals is to be free from painful emotions.	3.27	1.58	**.81**	–.06	.38

13. I work hard to keep out upsetting feelings.	3.34	1.47	**.76**	.07	.38

14. If I have any doubts about doing something, I just won’t do it.	2.97	1.46	**.40**	.32	.64

15. Pain always leads to suffering.	3.26	1.59	**.63**	–.04	.62


Correlation analyses between the subscales and the BEAQ total score showed significant positive correlations between the total score and the “Avoidance Tendency” subscale (*r* = .94, *p* < .01) and the “Emotional Unawareness and Inaction” subscale (*r* = .74, *p* < .01). Moreover, the two subscales were positively and significantly correlated with each other (*r* = .47, *p* < .01).

### Analysis of items, reliability and item-total correlations

At the item level, skewness ranged from –0.51 to 0.95 and kurtosis between –1.15 and –0.17; both were therefore acceptable for all items. Cronbach’s alpha for all BEAQ items was .83. Cronbach’s alpha was .83 for the first subscale and .58 for the second subscale. Analyses indicated that the removal of Item 6 could increase the alpha of the second subscale to .62 (and to .84 for the total scale). Problems with this item were also reported in other studies (Byllesby et al., 2020; Schaeuffele et al., 2022; Wardęszkiewicz & Holas, 2024). Therefore, it was decided to exclude Item 6 from the BEAQ total and subscale scores calculations and to not take it into account in subsequent analysis.

The BEAQ item-total correlations were all significant and ranged between .44 (item 3) and .72 (item 13). There was no floor or ceiling effect, since only a very small number of participants obtained extreme scores on the BEAQ and its subscales (see Table 3).

**Table 3:** Descriptive statistics and Cronbach’s alphas for the BEAQ total score and its two subscales.


	POSSIBLE SCORE RANGE	SCORE RANGE	MEAN	SD	LOWEST SCORE(FLOOR)	HIGHEST SCORE (CEILING)	CRONBACH’S ALPHA

BEAQ total score	14–84	18–84	45.20	11.89	0 (0%)	1 (0.2%)	.84

Subscale 1: Avoidance tendency	10–60	12–60	34.29	9.28	0 (0%)	1 (0.2%)	.83

Subscale 2: Emotional unawareness and inaction	4–24	4–24	10.91	4.15	17 (2.9%)	2 (0.3%)	.62


### Convergent and discriminant validity

After assumption checks were conducted, Pearson correlations were calculated to evaluate the BEAQ’s convergent and discriminant validity. The correlations between the BEAQ scores and its subscales with other convergent and discriminant measures are reported in Table 4. Correlations were examined between the BEAQ total score (BEAQ-Tot), the “Avoidance Tendency” (AT) and “Emotional Unawareness and Inaction” (EUI) subscales and life satisfaction (SWLS), psychological flexibility (AAQ-II), positive affects (PANAS-P), negative affects (PANAS-N), anxiety (HADS-A), depression (HADS-D), and personality traits extraversion (BFI-E), agreeableness (BFI-A), conscientiousness (BFI-C), neuroticism (BFI-N) and openness (BFI-O) (see Supplementary Material Section C for a description of the scores on the different scales used to assess the convergent and discriminant validity of the BEAQ).

**Table 4:** Correlations between BEAQ, its subscales and convergent and discriminant measures.


	BEAQ-TOTAL SCORE	SUBSCALE 1: AVOIDANCE TENDENCY	SUBSCALE 2: EMOTIONAL UNAWARENESS AND INACTION	AAQ-II

SWLS	–.29**	–.24**	–.31**	.62**

AAQ-II	–.51**	–.45**	–.47**	1

PANAS-PA	–.34**	–.25**	–.41**	.52**

PANAS-NA	.29**	.24**	.29**	–.62**

HADS-A	.32**	.28**	.29**	–.63**

HADS-D	.35**	.29**	.36**	–.63**

BFI-E	–.13**	–.07	–.21**	.27**

BFI-A	–.11**	–.09*	–.12**	.19**

BFI-C	–.19**	–.07	–.38**	.21**

BFI-N	.19**	.15**	.21**	–.56**

BFI-O	–.16**	–.12**	–.18**	.10*


Note. BEAQ = Brief Experiential Avoidance Questionnaire; SWLS: Satisfaction With Life Scale; AAQ-II: Acceptance and Action Questionnaire-II; PANAS-PA: Positive and Negative Affect Schedule-Positive Affect; PANAS-NA: Positive and Negative Affect Schedule-Negative Affect; HADS-A: Hospital Anxiety and Depression Scale-Anxiety; HADS-D: Hospital Anxiety and Depression Scale-Depression; BFI-E: Short Form of the Big Five Inventory-Extraversion; BFI-A: Short Form of the Big Five Inventory-Agreeableness; BFI-C: Short Form of the Big Five Inventory-Conscientiousness; BFI-N: Short Form of the Big Five Inventory-Neuroticism; BFI-O: Short Form of the Big Five Inventory-Openness. *Correlation significant at *p* < .05. **CorrelationDans Elsevier eBooks significant *p* < .01.

The scores on the BEAQ-Tot and each of the subscales were negatively correlated with satisfaction with life (respectively –.29, –.24 and –.31), psychological flexibility (respectively –.51, –.45 and –.47) and positive affects (respectively –.34, –.25 and –.41), indicating that higher levels of EA were associated with lower levels of satisfaction with life, psychological flexibility and positive affects. All correlations were weak to moderate in strength. The scores on the BEAQ-Tot and each of the subscales were weakly and positively correlated with negative affects (respectively .29, .24 and .29), anxiety (respectively .32, .28 and .29), and depression (respectively .35, .29 and .36), indicating that higher levels of EA were associated with higher levels of negative affects, anxiety and depression.

Regarding the relations between the BEAQ and the personality traits measured by the BFI-10, weak positive correlations were observed between the BEAQ-Tot, its subscales and neuroticism (respectively .19, .15 and .21). Moreover, the BEAQ-Tot and its subscales were weakly negatively correlated with openness (respectively –.16, –.12 and –.18) and agreeableness (respectively –.11, –.09 and –.12). The BEAQ-Tot and its EUI subscale were also negatively correlated with extraversion (respectively –.13 and –.21) and conscientiousness (respectively –.19, and –.38). However, there was no correlation between the AT subscale and these personality traits. Note that the EUI subscale of the BEAQ was more strongly correlated with conscientiousness than was the BEAQ-Tot. Therefore, higher levels of EA, and particularly higher emotional unawareness and inaction, were related to lower levels of conscientiousness.

Overall, the BEAQ total score and its two subscales were more strongly correlated with the AAQ-II than with measures of neuroticism/negative affectivity and mental health disorders. However, the AAQ-II presented stronger correlations with measures of mental health disorders and negative affectivity (PANAS-NA) than with the BEAQ and its subscales (see Table 4).

## Discussion

This study aimed to validate the BEAQ in French in a sample of non-clinical adults. The French version of the scale was developed using the translation–back-translation procedure (Vallerand, 1989). The results of the administration of the original and translated versions of the scale to a sample of bilingual participants two weeks apart showed that there was no significant difference between the English and the French versions of the BEAQ. In addition, the BEAQ showed good test-retest reliability at two-week intervals.

The French version of the BEAQ was then administered to a new sample to study its factor structure and convergent and discriminant validity. Confirmatory factor analyses did not support a one-dimensional structure for the BEAQ. This was also the case in other BEAQ validation studies (Chinese: Cao et al., 2021; German: Schaeuffele et al., 2022; Polish: Wardęszkiewicz & Holas, 2024). An exploratory factor analysis revealed a two-factor structure. The first factor comprised 10 items and was labeled “Avoidance Tendency”. It focused on the unwillingness to be in contact with unpleasant emotions, sensations and thoughts and the cognitive and behavioral avoidance strategies put in place to avoid these experiences. The second factor included five items and was labeled “Emotional Unawareness and Inaction”. This factor related to the lack of awareness or denial of internal experiences as well as the tendency to postpone or abandon important tasks in response to unpleasant internal experiences. The two subscales showed strong positive correlations with the BEAQ total score and were positively correlated with each other.

The French version of the BEAQ presented good internal consistency, similar to that of the original version of the tool. It should be noted, however, that one of the two subscales had a Cronbach’s alpha of less than .70. This could be due to the small number of items in the second factor compared to the first factor, since Cronbach’s alpha increases with the number of items in a scale (Tavakol & Dennick, 2011; Ursachi et al., 2015). Removing Item 6, the only one taken from the MEAQ “Distress Endurance” subscale, could increase the alpha (although it still did not reach the .70 threshold). A similar phenomenon was also observed in the Polish BEAQ validation study (Wardęszkiewicz & Holas, 2024). Problems with Item 6 were also noted in the German validation study (KMO < .60) (Schaeuffele et al., 2022) and another study conducted on a clinical sample (Byllesby et al., 2020). Consequently, the authors of the German and Polish versions suggested removing Item 6 from the scale. Given that removing this item could increase the Cronbach’s alpha of the second subscale of the BEAQ-French, we also suggest keeping only a 14-item version of the tool (i.e., without the Item 6).

The French version of the BEAQ presented satisfactory convergent and discriminant validity. BEAQ total and subscale scores were negatively correlated with psychological flexibility, life satisfaction and positive emotions while they were positively correlated with negative emotions, anxiety, and depression. This is consistent with previous studies showing EA is involved in the onset and maintenance of anxiety and mood disorders but is also associated with lower satisfaction with life and psychological flexibility, of which it is a specific aspect (Den Ouden et al., 2020; Gámez et al., 2014; Spinhoven et al., 2014). Regarding the relations between the BEAQ and personality traits, the BEAQ total and subscale scores were weakly and positively correlated with neuroticism. Small to moderate negative relationships were also observed between other personality traits and BEAQ total and subscale scores. It is interesting to note that the “Emotional Unawareness and Inaction” subscale was more strongly correlated with conscientiousness than the total BEAQ score was with this personality trait. This is theoretically relevant since conscientiousness relates to *“individual differences in the degree of organization, persistence, and motivation in goal-directed behavior”* and the “Emotional Unawareness and Inaction” subscale includes the tendency to postpone or abandon important tasks in response to unpleasant internal experiences (Stoeber et al., 2009, p. 364). Importantly, the BEAQ total and subscale scores were more strongly correlated with the AAQ-II than with measures of neuroticism, negative affect and psychopathology, while the AAQ-II scores were more strongly correlated with measures of negative affect (PANAS-NA) and psychopathology. This is consistent with previous studies’ results and indicates that the BEAQ is more appropriate than the AAQ-II as a measure of EA (Gámez et al., 2014; Gámez et al., 2011; Rochefort et al., 2018). Still the AAQ-II can be very useful, as it appears to be a better indicator of mental health problems since its scores are more strongly correlated with mental health problems than the BEAQ. Further research is needed to determine more precisely what it assesses.

Overall, the results indicate that the BEAQ exhibits good psychometric properties and can be used as a specific measure of EA. However, it is important to underline certain limitations of this scale. The first two relates to the fact that, as it has already been mentioned, the BEAQ is not one-dimensional as it was initially presented and that it presents problems with Item 6. As explained in the introduction, to create the BEAQ, Gámez et al., (2014) carried out an exploratory factor analysis on the MEAQ items and they selected the ones with high loadings on a single common factor. They also ensured that each MEAQ subscale was represented by at least one item in the final shorter scale. However, once the items of the shorter version were selected, the authors did not carry out a confirmatory factor analysis to demonstrate the adequate fit of a one-factor structure, which may explain why several other studies do not support the one-dimensional structure initially presented (Borgogna et al., 2023). Another limitation of the scale is that it is based on a definition of EA which is very close (Borgogna et al., 2023), but different from that of Hayes et al., (1996): *“the tendency to avoid (e.g., escape, control, suppress, modify, or not accept) the experience of negative affective (but not clearly dangerous) states”* (Gámez et al., 2011, p. 694). This definition places emphasis on negative affective states, which is not the case with the widely used definition of Hayes et al. (1996). Finally, like other measures of EA, the BEAQ assesses EA as a generalized trait. This has recently been criticized in a systematic review (see Akbari et al., 2022) which highlights the importance of assessing EA as a *“dynamic process, which varies as context varies”* (Akbari et al., 2022, p. 76). Despite these limitations, the BEAQ appears to be a short and interesting tool for studying EA, given its good internal consistency, test-retest reliability and its satisfactory discriminant validity.

The French version of this tool will provide health professionals and researchers with a fast and reliable tool to measure the effectiveness of their treatment. Indeed, recent research suggests that the BEAQ could detect changes in EA levels during and after treatment and therefore highlights a certain sensitivity of the BEAQ in measuring changes in EA levels over time (Schaeuffele et al., 2022; Schuman-Olivier et al., 2023). This is an important issue because several third-wave clinical interventions target this psychological process, but in the absence of a reliable tool to measure it, it is difficult to assess the effectiveness of these treatments in comparison to other, better-established treatments. Additionally, so far the AAQ-II has been the most popular measure of EA but evidence is accumulating to show that it is actually a measure of psychological distress or negative affect (Rochefort et al., 2018; Wolgast, 2014). This constitutes a bias affecting the results of previous research on EA if this process was measured with the AAQ-II. New tools for measuring EA are necessary to determine if we are able to replicate previous studies’ results. In this sense, the BEAQ-French could help researchers continue studies using French-speaking samples on the role of EA in mental health problems.

The results of this study should be interpreted in light of certain limitations. First, once the scale was translated, a set of students were asked to rate the extent to which these items seemed clear or unclear, based on a binary scale. A qualitative approach would have been more suitable during this phase to study participants’ perceptions and interpretations of the wording of items, including those judged to be clear. Second, the sample in the third validation step comprised a majority of women and is therefore not representative of the general population. Third, the convergent validity of the BEAQ was assessed based on its correlation with the AAQ-II. Unfortunately, given that the AAQ-II may actually measure psychological distress rather than psychological flexibility, it is not possible to state that this study was truly able to assess the BEAQ’s convergent validity. Fourth, personality was evaluated using the BFI-10, whose subscales, with the exception of Neuroticism and Extraversion, presented a Cronbach’s alpha below .70. This raises concerns about the extent to which items belonging to different subscales of the BFI-10 actually assess the same characteristic. It should be noted, however, that the Neuroticism subscale was the most important one in the context of this research, since it was used to study the discriminant validity of the BEAQ, and the Cronbach’s alpha of this subscale was satisfactory. Last, but not least, this study was not pre-registered. While pre-registration of clinical trials is highly recommended and even mandatory in some countries, it is not required for other types of studies. The present research fell into the latter category. It would nevertheless have been important to pre-record the research protocol for transparency purposes. In the present study, all validation steps and analyzes carried out were planned before the beginning of the study and were implemented as planned.

Despite these limitations, our study relies on a strong validation protocol respecting guidelines for the translation and validation of scales and a large sample of non-clinical participants. It constituted a first study of the psychometric properties and the factor structure of the BEAQ-French. Further research is needed to determine whether the two-factor structure in the present study is found with other samples. Studies should also further examine the Item 6 of the scale. Even if we recommend not taking into it account when calculating the BEAQ scores, it would be interesting to keep the item in future validation studies in order to study it further. Future research should also evaluate the convergent validity of the BEAQ with scales other than the AAQ-II, such as the Mindfulness Attention and Awareness Scale (MAAS; Brown & Ryan, 2003) which is a measure of mindfulness or the White Bear Suppression Inventory (WBSI; Wegner & Zanakos, 1994) which assesses the tendency to suppress unpleasant thoughts. It could also be interesting to examine the extent to which the two subscales of the BEAQ might be differently related to certain mental health problems or psychological constructs. Finally, future research should study the BEAQ in patient samples in order to establish norms for clinical populations and determine how the scores may differ in comparison to a non-clinical sample.

In conclusion, the French version of the BEAQ seems to be a fast and reliable tool to assess EA. The analyses support a two-factor rather than a one-factor structure of the scale, with one factor centered on avoidance and the other on emotional unawareness and inaction. The results of the present research also indicate keeping only a 14-item version and excluding Item 6 from the scale. Further research is needed to confirm this two-factor structure and to assess more precisely the convergent validity of the BEAQ.

## Data Accessibility Statement

The datasets associated to the present study are available from the corresponding author upon reasonable request.

## Additional File

The additional file for this article can be found as follows:

10.5334/pb.1256.s1 Supplementary Material. Section A, B and C.
