# Supplementary material for: The Brief Experiential Avoidance Questionnaire: Validation of the French Version in Non-clinical Adults
Source: Psychol Belg. 2024 Oct 4;64(1):152–65. doi: 10.5334/pb.1256 (PMC11451563; doi:10.5334/pb.1256)
Supplement: Supplementary Material. — Section A, B and C. [file pb-64-1-1256-s1.pdf]

Supplementary Material

A. French version of the Brief Experiential Avoidance Questionnaire

Veuillez indiquer dans quelle mesure vous êtes d'accord ou non avec chacune des propositions suivantes :

1: Fortement en désaccord 2: Modérément en désaccord 3: Légèrement en désaccord

4: Légèrement d'accord 5: Modérément d'accord 6: Fortement d'accord

	1	2	3	4	5	6
1. La clé d'une vie heureuse, c'est de ne jamais ressentir de la douleur.						
2. Je m'échappe rapidement de toute situation qui me fait me sentir mal à l'aise.						
3. Lorsque des souvenirs désagréables me viennent, j'essaie de les chasser de mon esprit.						
4. Je me sens déconnecté.e de mes émotions.						
5. Je ne fais jamais rien avant d'y être absolument obligé.e.						
6. La peur et l'anxiété ne m'empêcheront pas de faire quelque chose d'important.						
7. Je renoncerais à beaucoup de choses pour ne pas me sentir mal.						
8. Je fais rarement quelque chose s'il y a une chance que cela me contrarie.						
9. Il m'est difficile de savoir ce que je ressens.						
10. J'essaie de repousser le plus loin possible les tâches désagréables.						
11. Je fais tout ce qui est possible pour éviter les situations inconfortables.						
12. Un de mes objectifs principaux est de ne pas ressentir d'émotions désagréables.						
13. Je tente à tout prix d'éviter les sentiments qui me perturbent.						
14. Si j'ai le moindre doute sur ce que je dois faire, je ne le fais pas.						
15. La douleur conduit toujours à la souffrance.						

B. Scree plot

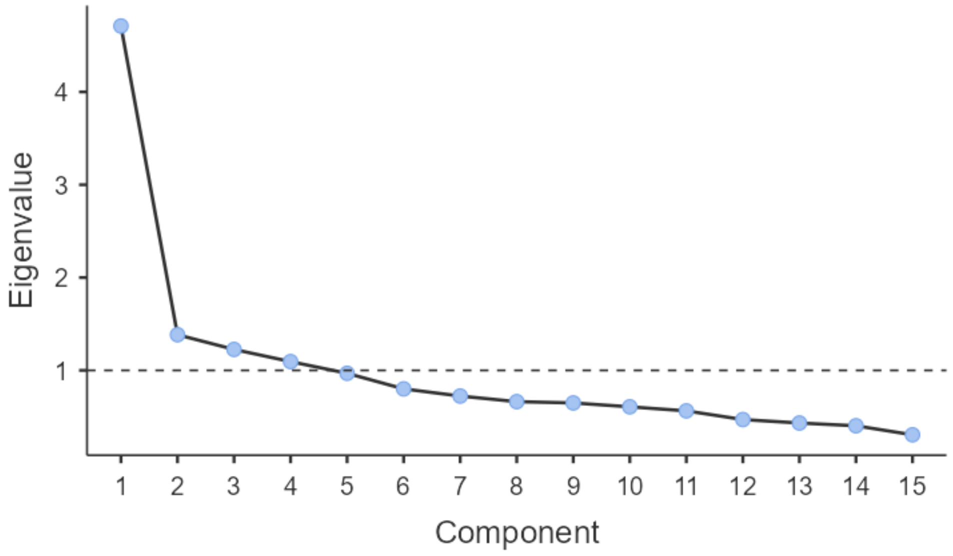

Figure S1. Scree plot

C. Descriptive analysis of the scores on the different scales used to assess the convergent and discriminant validity of the BEAQ

Table S1.

Scores on the different scales used to assess the convergent and discriminant validity of the BEAQ

	SWLS	AAQ-II	PANAS-PA	PANAS-NA	HADS-A	HADS-D	BFI-E	BFI-A	BFI-C	BFI-N	BFI-O
Mean	23,15	45,87	31,54	22,39	9,06	5,75	6,35	6,76	7,92	6,61	7,23
SD	6,795	10,123	7,548	8,510	4,312	3,794	2,398	1,866	1,803	2,551	1,984
Max.	35	69	48	49	21	20	10	10	10	10	10
Min.	5	19	10	10	0	0	2	2	2	2	2
Skewness	-,518	-,244	-,172	,698	,350	,762	-,127	-,302	-,553	-,239	-,263
Kurtosis	-,543	-,568	-,560	-,233	-,481	,343	-,981	-,286	-,372	-1,143	-,815

Note. BEAQ = Brief Experiential Avoidance Questionnaire ; SWLS : Satisfaction With Life Scale ; AAQ-II : Acceptance and Action Questionnaire-II ; PANAS-PA : Positive and Negative Affect Schedule-Positive Affect ; PANAS-NA : Positive and Negative Affect Schedule-Negative Affect ; HADS-A: Hospital Anxiety and Depression Scale-Anxiety; HADS-D: Hospital Anxiety and Depression Scale-Depression; BFI-E: Short Form of the Big Five Inventory-Extraversion; BFI-A: Short Form of the Big Five Inventory-Agreeableness; BFI-C: Short Form of the Big Five Inventory-Conscientiousness; BFI-N: Short Form of the Big Five Inventory-Neuroticism; BFI-O: Short Form of the Big Five Inventory-Openness
